# Perinatal genetic diagnostic yield in a population of fetuses with the phenotype arthrogryposis multiplex congenita: a cohort study 2007–2021

**DOI:** 10.1038/s41431-025-01848-3

**Published:** 2025-04-07

**Authors:** Arda Arduç, Johanna I. P. De Vries, Maria B. Tan-Sindhunata, Quinten Waisfisz, Eva Pajkrt, Ingeborg H. Linskens

**Affiliations:** 1 Department of Obstetrics and Gynecology, Amsterdam UMC, University of Amsterdam, Amsterdam, The Netherlands; 2 Amsterdam Reproduction and Development, Amsterdam, The Netherlands; 3 Amsterdam UMC Expertise Center FADS and AMC, Amsterdam, UMC the Netherlands; 4 Department of Human Genetics, Amsterdam UMC, University of Amsterdam, Amsterdam, The Netherlands

**Keywords:** Echocardiography, Genetic testing

## Abstract

Arthrogryposis multiplex congenita (AMC) presents challenges for prenatal detection due to its heterogeneous etiology, onset, and phenotypical manifestations. This study aims to describe the genetic diagnostic yield in a population of fetuses with detailed phenotypic description over a 15-year period (2007–2021) at the Fetal Medicine Unit of Amsterdam UMC, the Netherlands. The fetal and neonatal phenotypes were classified into three clinical AMC Groups, with the exception that Groups 1 and 2 were combined in the prenatal classification. Group 1 involves limb involvement primarily, Group 2 includes musculoskeletal involvement plus other system anomalies, and Group 3 involves musculoskeletal involvement with central nervous system disability, lethality, fetal akinesia deformation sequence, and/or intellectual disability. The cohort consisted of 64 consecutive cases, 13 in Groups 1 + 2 and 51 in Group 3. Perinatal genetic testing occurred in all cases: prenatally in 56 of the 64 (88%), postnatally in 36 of the 64 (56%), and combined testing in 28 of the 64 cases (44%). The overall genetic diagnostic yield was 28% (18/64), and it increased over the 5-year period from 14% to 50%. Whole exome sequencing had the highest yield (41.7%). The yield per phenotype was 30.8% (4/13) for AMC Group 1 + 2 and 27.4% (14/51) for AMC Group 3. Detailed fetal phenotyping and perinatal genetic testing in all cases showed improved diagnostic yield over time, likely due to the introduction of Next-generation sequencing-based tests. The availability of stored DNA will be beneficial for future investigations since further improvements in genetic testing possibilities are expected.

## Introduction

Prenatal detection of arthrogryposis multiplex congenita (AMC) remains an ongoing challenge. AMC is a rare congenital condition characterized by multiple contractures affecting various joints [1]. Detecting this condition before birth is complicated due to a variety of factors.

Firstly, the etiology of AMC varies from genetic and environmental to unknown factors [1, 2]. Secondly, the onset of AMC varies from the first through the third trimester of pregnancy. Thirdly, the expression of the spectrum AMC ranges from mild to severe. Postnatally, Hall et al. initiated the classification of AMC for therapeutic and prognostic purposes into three main groups dependent on involvement: Group 1 with primarily limb involvement, Group 2 with musculoskeletal involvement plus other system anomalies, and Group 3 with musculoskeletal involvement, central nervous system dysfunction and/or intellectual disability and/or lethality [3, 4]. Fetal akinesia deformation sequence (FADS) is one of the lethal forms of AMC with multiple contractures, abnormal facial profile, small thorax, and motility deterioration during pregnancy and after birth with a variety of causes [4]. In line, this classification has been used prenatally to differentiate between the various phenotypes of AMC [5].

Management of all groups of AMC requires a multidisciplinary team of experts for perinatal parental counseling, aiming for care before and around birth, and throughout life. The quality of life of children within Groups 1 and 2 is dependent on the cause of AMC. Two studies showed that, in general, these children have an acceptable level of independence and an overall high quality of life [6, 7]. The outcome and prognosis of children in Group 3 is also dependent on the etiology, however, in case of FADS the majority will die perinatally [8, 9].

The prenatal detection of AMC by sonographic examination has shown little improvement over time. A study showed a prenatal detection of AMC in 28 of 107 children (26%) with Amyoplasia born after 1990 [10]. In 2019, Dahan-Oliel et al. reported a prenatal detection of 53% (21/40) among infants with confirmed AMC Groups 1 and 2 [11]. In addition, a prenatal detection of 37% was reported by Lemin et al. in 2024 within a cohort of 301 infants with AMC Groups 1 and 2 [12]. The integration of serial motor assessment of the fetus with structural anomaly scanning and evaluation by a multidisciplinary team further enhanced prenatal detection to 100% in a high-risk population with AMC Groups 1–3 (*n* = 66) [13].

Not all causes of AMC can be explained by genetic abnormalities. Currently, more than 400 different genes have been associated with AMC [2]. Advances in perinatal genetic testing have evolved over time. Karyotyping for aneuploidies has been available since the 1960s [14]. Subsequent innovations have introduced fluorescence in situ hybridization, FISH (1990s), quantitative fluorescence-polymerase chain reaction, QF-PCR (2000), and multiplex ligation-dependent probe amplification, MLPA (2002), enabling the identification of common aneuploidies in chromosomes 13, 18, and 21 [15–18]. Chromosomal microarrays, in use since 1995, marked a leap forward, allowing for the detection of DNA deletions and duplications [19]. Sanger sequencing, a single-gene test widely used since 1977, has been largely replaced by next-generation sequencing (NGS) due to its greater speed and ability to sequence multiple genes simultaneously [20]. The use of whole exome sequencing (WES) has shown an increase since 2009 [21–23].

In this study, we aim to present the phenotype and genotype in a 15-year cohort of consecutive fetuses with prenatally suspected and postnatally confirmed AMC. The phenotypic descriptions were prenatally based on (serial) sonographic structural anomaly scans, with systematic or descriptive motor assessment. The postnatal phenotype was based on external examinations with or without neurological examinations and with or without post-mortem examination in case of termination of pregnancy or neonatal death. The performed genetic tests and the overall genetic diagnostic yield of the 15-year period were evaluated, as well as the yield per each 5-year period. This study includes recommendations for genetic testing in case of prenatally suspected AMC, ensuring alignment with the available resources and capabilities in a hospital setting.

## Subjects and methods

### Inclusion and exclusion criteria

Inclusion criteria encompassed cases meeting the following criteria: prenatal suspicion of at least two contractures in different anatomical regions (e.g., a combination of contractures in ankle, knee, hip, fingers, wrist, elbow, shoulder) or contracture(s) in a single region with FADS-associated anomalies like facial anomalies, webbing, cerebral anomalies, hydrops, hypo-/akinesia and/or polyhydramnios with or without a family history of FADS/AMC. The suspicion of multiple contractures had to be confirmed in the postnatal period by at least a physical examination of the newborn or post-mortem evaluation in case of intrauterine fetal death or termination of pregnancy. Postnatal evaluations occur directly after birth by a neonatologist in case of live birth, and by an obstetrician, clinical geneticist, and/or pathologist in case of death. Excluded were those who did not fulfill the above-mentioned criteria, for example, contractures in one anatomic region (e.g., isolated clubfeet).

### Study population

In the Netherlands, pregnant women with suspected structural anomalies during a routine mid-trimester fetal ultrasound examination receive targeted anomaly ultrasound examination in a tertiary Fetal Medicine Unit [24]. In line, all cases underwent (serial) targeted anomaly scans in one of our two Fetal Medicine Units of the tertiary Amsterdam University Medical Center (UMC) in the Netherlands between January 1, 2007, and December 31, 2021. The Medical Ethical Committee of the Amsterdam UMC granted approval under reference number W21_361 # 21.401, and data were extracted at both UMC locations, VU University Medical Center (VUmc) and Academisch Medisch Centrum.

### Data collection

For the description of the prenatal and postnatal phenotype and genotype, data were collected using the Fetal Medicine Units’ Astraia database for ultrasound examinations, EPIC (Electronic Portfolio of International Credentials) for patient charts, and Genesis (GENetic Estimation and Inference in Structures samples) for genetic results. Also, it was evaluated if DNA was stored for potential future DNA re-assessment. Moreover, data extraction from location VUmc was supported by the perinatal database maintained by the Amsterdam UMC Expertise Center for FADS and AMC [25]. This expertise center on rare diseases has been acknowledged by the Ministry of Health, Welfare, and Sport since 2015, first at location VUmc and when both Amsterdam UMC locations merged, continued at location Amsterdam Medisch Centrum in 2021.

### Phenotypic and genotypic classification

Postnatally, the phenotypes were divided into the three AMC Groups according to Hall et al. [4]. However, prenatally, AMC Group 1 and 2 were merged due to sonographic limitations in distinguishing between AMC Group 1 and 2. For instance, it is challenging to detect the more pronounced muscle atrophy characteristic of Group 1 compared to Group 2 by using ultrasound. Additionally, since intellectual disability cannot be detected through ultrasound examinations, the phenotypes of Hall et al. were modified for the prenatal period as follows:

- AMC Group 1 + 2: primary limb involvement + musculoskeletal involvement plus other system anomalies;
- AMC Group 3: musculoskeletal involvement with central nervous system involvement and/ or lethality within the spectrum of FADS (expanding contractures or abnormal/ worsening fetal movements or FADS-related anomalies).

In cases where the prenatal or postnatal differential diagnosis had overlapping phenotyping Groups 2 and 3, the phenotype was classified as Group 3 to emphasize the wide spectrum of AMC from milder to severe expressions.

The fetal structural anatomy in all cases was examined by targeted anomaly scans, and fetal movements by systematic motor assessment (sMA) or descriptively. At location VUmc, the majority of the fetuses underwent sMA. Details concerning the organization of the 7-step care pathway and the sMA evaluation can be read in the care pathway, which was designed by a multidisciplinary team of our expertise center for AMC [25]. The 15-minute motor assessment is conducted before a gestational age of 24 weeks to distinguish between the milder types of AMC and its most severe form (FADS), given the legal limit for pregnancy termination in the Netherlands. During fetal movement recording, we evaluate differentiation (into movement patterns), quantity (frequency of general movements involving all limbs, trunk, and head), and quality (of general movements, isolated arm- and leg movements concerning variation in amplitude, speed, joint participation, and direction). At the Academisch Medisch Centrum, the movements were mainly described descriptively. Movements were considered abnormal if the sMA, evaluating three aspects, differentiation (D) into specific movement patterns, qualitative (QL), and quantitative performance (QN), during a sufficient long age-related observation period, revealed at least an abnormal qualitative performance [13, 25]. Movements evaluated descriptively were considered abnormal if the sonographer documented that the movements were abnormal, reduced, or absent.

### Prevalence calculation

The prevalence of AMC was estimated per 10,000 pregnancies in the North-West region of the Netherlands. The number of women receiving routine mid-trimester fetal ultrasound examination in this region was derived from not published, but digitized yearly audit files per prenatal ultrasound examination unit. Moreover, we estimated the number of cases within the same region in which a targeted anomaly scan was either not performed, the mid-trimester fetal ultrasound examination did not lead to a prenatal referral, or in case the anomaly was not identified prenatally. These children were postnatally treated by pediatric orthopedic surgeons and/or physiatrists and were described separately in this study. We used this number to identify missing prenatal cases with the AMC phenotype.

### Genetic testing

The type and number of tests that were performed after counseling by the clinical geneticist and depending on parents’ request, were examined. Genetic tests that were used included karyotyping, rapid aneuploidy testing (FISH, QF-PCR, or MLPA), chromosomal microarray, single gene testing, panel (various panels are available e.g., targeted Arthrogryposis panel and WES based FADS panel), and WES. WES-based copy number variation (CNV) analysis was not implemented during the study period. Gene panels were updated yearly during the study period. The genes included in the latest FADS panel used in the study period are listed in Supplementary File 2. Findings per genetic test were presented for the prenatal phenotypes AMC Group 1 + 2 and for AMC Group 3. Additionally, the analysis is performed for the 15-year period as well as the three consecutive 5-year periods. For this study, all genotypic and phenotypic results were re-examined, and genetic evaluation was updated with the present literature.

The results of prenatal genetic testing in women with more than one affected pregnancy with the same phenotypes and/or genotypes were initially assessed for each pregnancy individually, but subsequently, the results were combined to form one case.

### Statistics

Changes in the prevalence of genetic tests and yield were compared by descriptive statistics in Excel and Microsoft 356 A3 for faculty. The genetic overall yield is evaluated in percentages. A chi-square analysis was performed to evaluate the differences in genetic diagnostic yield between the three 5-year intervals. A *p*-value < 0.05 was considered statistically significant.

## Results

### Characteristics

In 64 women with a total of 81 pregnancies, the inclusion criteria of their fetuses fulfilled the criteria of AMC, suspected by targeted anomaly scan and confirmed postnatally. The distribution of the phenotypes in the 64 women consisted of 13 with the prenatal phenotype within Group 1 + 2 and 51 within Group 3. The pregnancy outcomes are summarized in Table 1A and Supplementary Table 1A. There were eight women (cases 2008-3, 2009-3, 2010-1, 2012-6, 2012-10, 2015-2, 2017-7, 2020-1) with more than one pregnancy (total n = 25), showing a similar prenatal phenotype and all within Group 3.

**Table 1:** A: Description of the prenatal and postnatal phenotype of 18 fetuses with AMC, classified according to Hall et al. [1]; B: Description of genotype based on perinatal genetic tests of 18 fetuses with AMC identified in the prenatal period at Amsterdam UMC during the period 2007–2021.

A																		
CASE	PRENATAL												POSTNATAL					
	Pregnancies with same phenotype	Consanguinity	Sonographic phenotype															
			Detection of the contractures (weeks)	Contractures CAL=arms and Legs	Enlarged nuchal translucency/fold	Hydrops	Pterygia	Amniotic fluid volume N = normal P = polyhydramnios O = oligohydramnios	CNS malformation	intrauterine growth restriction	Systematic motor assessment. If yes: differentiation(D),Quality (QL), quantity (QN): normal (nl)/ abnormal (abn) If no: descriptively	AMC group	Outcome A= alive at birth, IUFD = intrauterine fetal death, TOP = termination of pregnancy, N = neonatal death Gestational age at birth in weeks	Contractures CAL = arms and Legs	Additional anomalies CAL = arms and Legs	Autopsy	Age at last clinical evaluation. DPN = direct postnatal	AMC group
2008-1*	1	−	20 + 3	CAL: wrist, clubfoot Other: micrognathia	−	−	−	N	−	−	Yes 2x D, QL, QN nl	1 + 2	A, 40 + 4	CAL: fingers, wrist, clubfoot, clubfeet	Aortic root dilatation, pectus carinatum, kyphoscoliosis, normal cognitive development	−	16 years, diagnosis at age of 4	2
2008-3*, ***	5	+	13 + 3 12 + 2 10 + 5 10 + 6 11 + 0	CAL: wrists and clubfeet	+	+	+	N	−	−	No Absent fetal movements	3	TOP, 14 + 0 TOP, 12 + 6 TOP, 11 + 0 TOP, 11 + 6 TOP, 11 + 6	CAL: wrists and clubfeet	Cystic hygroma, lung hypoplasia, webbing elbows/knees − − − −	+ − − − −	DPN DPN DPN DPN DPN	3
2010-1*	3	−	21 + 3 18 + 5 13 + 0	CAL: wrists, knees and clubfeet	+	+	−	P	−	−	Yes Only in 2^nd^ pregnancy: D, QL, QN abn	3	IUFD, 31 + 3 TOP, 23 + 6 IUFD, 13 + 3	CAL: wrists, knees and clubfeet	− − Hydrops	+ + +	DPN DPN DPN	3
**2012-3**	1	+	20 + 5	CAL: hips, knees, elbows, shoulders, wrists and fingers	+	−	−	N	Ventriculomegaly, choroid plexus cysts	−	No Absent movements	3	TOP, 23 + 0	CAL: hips, knees, elbows, shoulders, wrists and fingers	Micro-retrognathia	+	DPN	3
2012-10*	2	−	20 + 5 12 + 6	CAL: elbows, wrists, knees, clubfeet Other: short legs	+	−	−	N	−	−	Yes D abn QL abn isolated arm and leg movements variation reduced, general movements variation reduced, no in- and decreasing activity, abrupt movements QN nl	3	TOP, 23 + 5 TOP, 15 + 0	CAL: elbows, wrists, knees, clubfeet Other: short legs	Retrognathia, hitchhiker thumbs −	+ −	DPN DPN	3, diastrophic dysplasia
**2015-2**	2	+	13 + 6 20 + 1	1st pregnancy CAL: legs and arms 2^nd^ pregnancy CAL: wrists, clubfeet Heart: VSD	+	−	−	N	−	−	No Absent movements	3	TOP, 14 + 6 TOP, 21 + 5	1st pregnancy CAL: legs and arms 2^nd^ pregnancy CAL: wrists, clubfeet	Webbing Micrognathia, Coarctation of the aorta	+ +	DPN DPN	3, Nemaline myopathy-8 only in 2^nd^ pregnancy
2016-3*	1	−	19 + 5	CAL: flexed wrists, clubfeet, Other: short femur	−	−	−	N	−	+	Yes D nl, QL of general movements abnormal (reduced participation in hands and feet), QN nl	1 + 2	A, 40 + 0 Died at age of 5 due to a septic shock after a RS virus pneumonia	CAL: flexed wrists, overlapping toes, clubfeet,	Other: feeding problems, bifid uvula, ptosis, small deepset eyes	−	5 years	2, Distal Arthrogryposis type 3 or 5
**2017-2**	1	−	20 + 1	CAL: extended knees and abducted hips	−	−	−	N	−	−	No Normal overall movements, no movements in knees	2	A, 41 + 1	CAL: extended knees, abducted hips, ankle and finger contractures	Short neck, small mouth, mild retrognathia, ptosis, scoliosis, nl cognitive development. Walking	−	7 years, diagnosis in 5th month	2, Distal Arthrogryposis type 5
2017-4*, **	1	−	14 + 5	CAL: elbows, wrists, clubfoot Heart: abnormal aortic arch	+	−	−	N	−	−	Yes 2x, D nl, AL abn, worsening over time, QN nl	3	N, 38 + 6 Died after 4 months due to respiratory insufficiency	CAL: fingers, elbows, wrists, clubfoot	Heart: cardiomyopathy (open ductus and abnormal aortic arch)	−	4 months	3
**2017-6**	1	−	18 + 4	Fingers bilateral	−	−	−	N	−	−	No Normal movements	2	A, 38 + 6	CAL: fingers, elbows, hips	Heart : small VSD Normal cognitive development. Walking independently.	−	8 years	2
**2018-4**	1	−	19 + 5	CAL: arms, legs and clubfeet Other: empty stomach	+	+	−	P	−	−	No Absent movements	3	TOP, 22 + 3	CAL: arms, legs and clubfeet	Micrognathia, hypertelorism	−	DPN	3
2019-2*	1	−	19 + 5	CAL: clubfeet Other: open mouth.	+	+	−	N	−	−	Yes 2x D nl, QL abn and worsening over time, QN nl	3	N, 39 + 0 Child died at 2 months, respiratory insufficiency	CAL: wrist contractures and clubfeet	Floppy infant Other: retro/micrognathia, renal cysts	−	2 months	3
**2019-3**	1	−	19 + 4	CAL: overlapping fingers, fixed elbows, wrists, knees and clubfeet Other: short limbs, Small thorax, hypospadias	−	−	−	N	−	−	No Decreased movements	3	TOP, 21 + 1	CAL: overlapping fingers, fixed elbows, wrists, knees and clubfeet	Webbing elbows, cleft palate	−	DPN	2/3 Distal arthrogryposis type 3 or 5
2019-4*	1	−	20 + 3	CAL: overlapping fingers, clubfeet Other: heart: VSD, micrognathia, omphalocele	−	−	−	P	Blake’s pouch cyst	−	No Normal movements	3	TOP, 21 + 3	CAL: overlapping fingers, clubfeet	No cerebral anomalies	+	DPN	3
2020-1*	2	−	12 + 0 13 + 4	CAL: fixed arms, clubfeet	+	+	−	N	−	−	No Absent movements	3	TOP, 12 + 3 TOP, 14 + 3	CAL: fixed arms, clubfeet	− Webbing	− +	− DPN	3
**2020-5**	1	−	16 + 6	CAL: fingers, elbows, knees	+	−	−	N	−	−	No Absent fetal movements	3	TOP, 21 + 1	CAL: fingers, elbows, knees	Micrognathia, webbed neck	−	−	3
2020-8*	1	−	19 + 5	CAL: hands, clubfeet	−	−	−	N	−	−	Yes 2x, D, QL and QN abn	3	TOP, 21 + 5	CAL: hands, clubfeet	Micrognathia	+	DPN	3
2021-2*	1	−	19 + 3	CAL: Clubfeet Open mouth	−	−	−	N	−	−	Yes 2x. 19 wk D, QL and QN nl, 22 wk worsening: D nl, QL abn, absent variation in amplitude, speed, participation and direction, no in and decreasing activity only fluency, in isolated arm movements and general movements, QN nl	3	TOP, 22 + 5	CAL: Clubfeet Open mouth	Contractures wrists, elbows, fingers, hips and knees	−	DPN	2/3

B							
CASE	Genetic tests A karyotyping B rapid aneuploidy C single gene testing D chromosomal microarray E1 panel E2 WES E3 genome-wide linkage scan and Sanger sequencing in research setting Bold for test detecting the mutation	Pre/postnatal testing? Pr=prenatal Po = postnatal Pr/Po testing both pre- and postnatal	Inheritance DN = de novo AR = autosomal recessive	Nucleotide alteration, deduced protein change	ACMG criteria P = pathogenic LP = likely pathogenic	Aggregated Pathogenicity Prediction (Franklin)	MAF (according to gnomAD v4.1.0)
2008-1*	A, **C**, D	Pr/**Po**	DN	NM_004612.4(TGFBR1):c.1013 A > G p.(Asn338Ser) Chr9(GRCh37):g.101907053 A > G	LP: PM1, PM2, PM6, PP2, PP3	Deleterious (0.87)	−
2008-3*, ***	A, B,C, E1, **E3**	Pr/**Po**	AR (Homozygous confirmed in 4 affected sibs, both parents heterozygous)	NM_000540.3(RYR1):c.6721 C > T p.(Arg2241*) Chr19(GRCh37):g.38987106 C > T	P: PVS1, PM2, PM3, PP5	−	0.0001611 (no homozygous alleles)
2010-1*	A, B, **E2**	Pr/ **Po**	AR (2 affected sibs, compound heterozygous) Both parents and one healthy sib heterozygous, one healthy sib no carrier.	NM_000334.4(SCN4A):c.274-2 A > G p.? Chr17(GRCh37):g.62049832 T > C (maternal) NM_000334.4(SCN4A):c.3911_3912+1del p.(Lys1304Ilefs*17) Chr17(GRCh37):g.62022041_62022043del (paternal) Confirmed in all 3 fetuses	P: PVS1, PM2, PM3, PP1 P: PVS1, PM2, PM3, PP1	− −	− 0.00001812 (no homozygous alleles)
2012-3	A, **C**, D	**Pr/** Po	AR (Homozygous, both parents heterozygous)	NM_005592.4(MUSK):c.1724 T > C p.(Ile575Thr) Chr9(GRCh37):g.113547944 T > C	LP: PS3, PM2, PP1, PP3, PP5	Deleterious (0.87)	0.00001178 (no homozygous alleles)
2012-10*	A, B, **C**, D, E1	Pr/**Po**	AR (Compound heterozygous) Both parents heterozygous	NM_000112.4(SLC26A2):c.931 T > C p.(Cys311Arg) Chr5(GRCh37):g.149360087 T > C (paternal) NM_000112.4(SLC26A2):c.1957T>A p.(Cys653Ser) Chr5(GRCh37):g.149361113 T > A (maternal)	LP: PM2, PM3, PP3, PP5 P: PS3, PM1, PM2, PM3, PP1, PP3, PP5	Deleterious (0.87) Deleterious (0.86)	0.000002478 (no homozygous alleles) 0.0001121 (no homozygous alleles)
2015-2	B, D, E1, **E2**	Pr/**Po**	AR (homozygous, both parents heterozygous) Only in 2^nd^ pregnancy	NM_152393.4(KLHL40):c.360 C > A p.(Cys120*) Chr3(GRCh37):g.42727470 C > A	LP: PVS1, PM2	−	0.000006204 (no homozygous alleles)
2016-3*	B, D, **E2**	Pr/**Po**	DN	NM_022068.4(PIEZO2):c.2994 G > A p.(Met998Ile) Chr18(GRCh37):g.10762974 C > T	LP: PM2, PM5, PM6, PP5	Uncertain (0.57)	−
2017-2	B, D, **E2**	Pr/**Po**	AR (homozygous, both parents heterozygous)	NM_004826.4(ECEL1):c.1470 G > A p.(Trp490*) Chr2(GRCh37):g.233348162 C > T	P: PVS1, PM2, PM3, PP5	−	0.00002232 (no homozygous alleles)
2017-4*, **	B, **E1**	Pr/ **Po**	DN	NM_001005361.3(DNM2):c.1090 C > T p.(Arg364Cys) Chr19(GRCh37):g.10904493 C > T	LP: PM1, PM2, PM5, PM6, PP2, PP3	Deleterious (0.72)	−
2017-6	B, **D**	**Pr**	Maternal Mother and maternal grandfather both carrier of the duplication both affected with distal arthrogryposis	Arr[GRCh37] 6q24.3q25.2(145784222_153395582)x3 (gain of 7.6 Mb)			
2018-4	B, D, **E1**	**Pr**/Po	AR (Homozygous, both parents heterozygous)	NM_005592.4(MUSK):c.220 C > T p.(Arg74Trp) Chr9(GRCh37):g.113449410 C > T	LP: PM2, PM3, PP1, PP5	Uncertain (0.56)	0.000008059 (no homozygous alleles)
2019-2*	**E2**	**Po**	AR (Homozygous, both parents heterozygous)	NM_000287.4(PEX6):c.814_817dup p.(Val273Alafs*9) Chr6(GRCh37):g.42946073_42946076dup	P: PVS1, PM2, PP5	−	−
2019-3	B, C, D, E1, **E2**	Pr/ **Po**	DN	NM_022068.4(PIEZO2):c.8056 C > T p.(Arg2686Cys) Chr18(GRCh37):g.10671727 G > A	P: PS2, PM2, PM5, PP2, PP3, PP5	Deleterious (0.99)	−
2019-4*	B, D, **E2**	Pr/**Po**	AR (Compound heterozygous)	NM_024105.4(ALG12):c.200 C > G p.(Thr67Arg) Chr22(GRCh37):g.50307128 G > C (maternal) NM_024105.4(ALG12):c.437 G > A p.(Arg146Gln) Chr22(GRCh37):g.50304114 C > T (paternal)	LP: PM2, PM3, PM5, PP3 PS3, PM2, PM5, PP3, PP5	Deleterious (0.87) Deleterious (0.74)	− 0.0001233 (no homozygous alleles)
2020-1*	B, D, **E2**	**Po**	AR (2 affected sibs, both compound heterozygous)	NM_001164508.2(NEB):c.22205del p.(Asn7402Thrfs*11) Chr2(GRCh37):g.152381745del (maternal) NM_001164508.2(NEB):c.2014_2015del p.(Leu672Ilefs*30) Chr2(GRCh37):g.152548664_152548665del (paternal)	LP: PVS1, PM2 LP: PVS1, PM2	− −	− 6.197e−7 (no homozygous alleles)
2020-5	B, D, **E1**	**Pr**	AR (Compound heterozygous) Both parents heterozygous	NM_005199.5(CHRNG):c.397del p.(Ser133Profs*50) Chr2(GRCh37):g.233406130del (maternal) NM_005199.5(CHRNG):c.401_402del p.(Pro134Argfs*43) Chr2(GRCh37):g.233406134_233406135del (paternal)	P: PVS1, PM2, PM3, PP5 P: PVS1, PM2, PM3, PP5	− −	0.0001047 (no homozygous alleles) 0.00009046 (no homozygous alleles)
2020-8*	B, E1, **E2**	Pr/ **Po**	DN	NM_003289.4(TPM2):c.782 A > G p.(Tyr261Cys) Chr9(GRCh37):g.35683229 T > C	LP: PM2, PM6, PP2, PP3, PP5	Deleterious (0.86)	−
2021-2*	B, **E2**	Pr/ **Po**	AR (Compound heterozygous) Both parents heterozygous	NM_022068.4(PIEZO2):c.492+2 T > C p.? Chr18(GRCh37):g.10871249 A > G (maternal) NM_022068.4(PIEZO2):c.570 C > T p.(Gly190 = ) / r.spl? Chr18(GRCh37):g.10857132 G > A (paternal)	LP: PVS1, PM2 VUS: PM2	Deleterious (0.8) SpliceAI: Splice-Altering/strong (0.99) Deleterious (0.79) SpliceAI: Splice-Altering/strong (0.97)	− 6.503e−7

Identified in the prenatal period at Amsterdam UMC during the period 2007–2021. The genotype is presented in Table 1B.

* For cases that were seen at location VUmc.

** For cases that were published by Tjon et al. [13].

*** For one case that was published by McKie et al. [52].

Classification following ACMG Criteria [53].

*** Published by McKie et al. [52].

The distribution of the cases was 38 at location VUmc and 26 at location Academisch Medisch Centrum. The distribution of the fetal phenotype Groups 1 + 2 and 3 did not differ between both locations (*p* = 0.28). Motor evaluation was available in 100% at location VUmc of which 84% (32/38) by sMA and 16% (6/38) descriptively, and in 92% (24/26) descriptively at location Academisch Medisch Centrum. In the remaining 2 cases there were no descriptives on fetal movements reported.

### Prevalence

During the study period, a total of 485,000 pregnant women received a mid-trimester fetal ultrasound examination in the North-West of the Netherlands. The prevalence of AMC per 10,000 pregnancies with mid-trimester fetal ultrasound examination in North-West Netherlands was 1.7 (81 pregnancies of the 64 mothers /485,000). The pediatric orthopedic surgeons and physiatrists of Amsterdam UMC treated another four children with AMC who were not included in this prenatal cohort (one case with AMC in Group 1 and 3 in Group 2). They underwent routine mid-trimester fetal ultrasound examinations between 2007-2021 in the North-West region of the Netherlands. During this routine mid-trimester fetal ultrasound examination, twice, no anomalies were identified, and therefore, they were not referred to a Fetal Medicine Unit. The remaining two cases were referred because of isolated contractures (one clubfoot and one wrist contracture), but the targeted anomaly scans did not reveal other anomalies. Postnatally, examinations showed multiple contractures in all four children.

### Prenatal and postnatal phenotype

The phenotype and pregnancy outcomes of the fetuses with a genetic diagnosis are summarized in Table 1A and for fetuses without a genetic diagnosis in Supplementary Table 1A.

The fetal phenotypes AMC Group 1 + 2 were suspected in 13 cases. Postnatal confirmation occurred in all, with 6 in Group 1 and the remaining 7 in Group 2. The outcome was live-born children in 9 (69%) and termination of pregnancy in 4 (31%).

An AMC Group 3 phenotype was suspected in 51 cases. Postnatal confirmation occurred in all, with 49 cases classified in Group 3, and 2 into Group 2 (Table 1A and Supplementary Table 1A). Of these 51 cases with a total of 68 pregnancies, the outcome was 3 live-born children (4%), 46 (68%) terminations of pregnancy, 12 (18%) intrauterine fetal deaths, and 7 (10%) neonatal deaths.

### Genetic tests

**Fig. 1:**
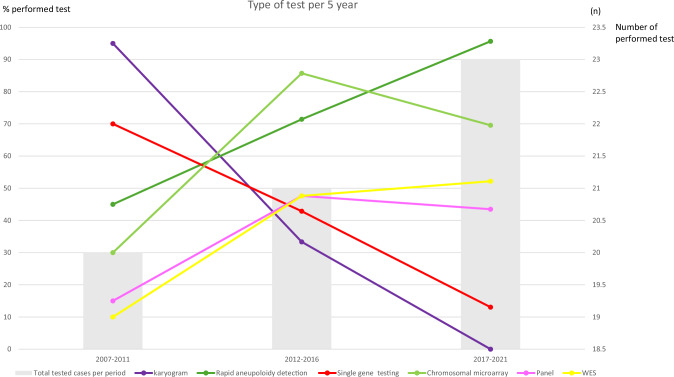
Genetic tests performed in a population of fetuses suspected of arthrogryposis multiplex congenita in a 15-year cohort, depicted per 5-year periods in numbers and percentages.

Perinatal genetic testing occurred in 100%. Prenatal genetic testing was performed in 88% (56/64). Postnatally, 56% (36/64) of the cases received first-time genetic testing or extended testing (Table 1B and Supplementary Table 1). Combined pre- and postnatally testing occurred in 28 of the 64 cases (44%). The total number of tests and percentages of tests that were conducted per 5-year period are depicted in Fig. 1. DNA samples from all cases have been stored and remain available for future re-assessment.

### Genotype

**Fig. 2:**
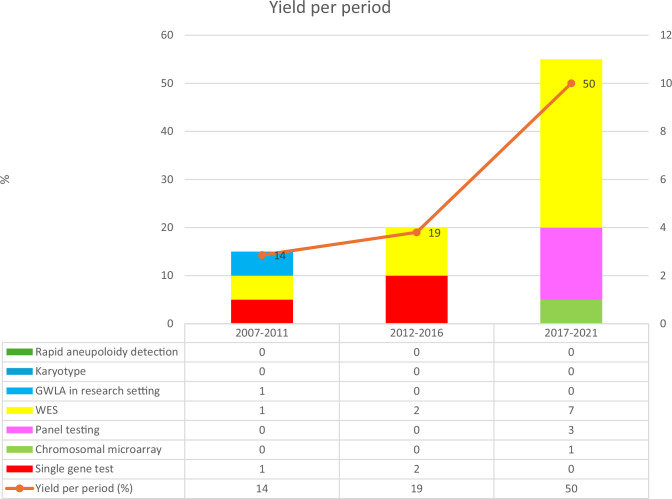
Perinatal genetic diagnostic yield in a cohort of 64 fetuses suspected of arthrogryposis multiplex congenita, depicted per 5-year periods in percentages (number of genetic abnormalities found per fetus). GWLA genome-wide linkage scan and Sanger sequencing in a research setting.

The overall genetic diagnostic yield for the study population was 28% (18/64). The overall genetic diagnostic yield per 5-year interval is shown in Fig. 2. An increase was seen in the genetic diagnostic yield with the performed tests between the three 5-year intervals (*p* = <0.00001), with the highest yield of 50% between 2017–2021. The genetic diagnostic yield per group and per test are summarized in Table 2. Panel testing and WES had the highest yield (13% and 41.7%). The yield per phenotype was 30.8% (4/13) for AMC Group 1 + 2 and 27.4% (14/51) for AMC Group 3. Variants were identified in the *ALG12*, *CHRNG*, *DNM2*, *ECEL1*, *KLHL40*, *MUSK* (2×), *NEB*, *PEX6*, *PIEZO2* (3×), *RYR1*, *SCN4A*, *SLC26A2*, *TGFBR1*, and *TPM2* genes. The variant in *RYR1* was identified through a genome-wide linkage scan and Sanger sequencing of the candidate gene. Additionally, genetic testing in this group revealed one maternally derived chromosomal abnormality, a microduplication 6q24.3q25.2. There are no cases that were diagnosed after re-analysis. Description of the genotype based on perinatal genetic tests is shown in Table 1B and Supplementary Table 1B.

**Table 2:** Genetic yield per test in a cohort of 64 women with fetuses suspected for AMC groups 1, 2, and 3 during a 15-year period 2007–2021.

Group(s) with AMC - AMC 1,2,3 - AMC 1 + 2 - AMC 3	Genetic test(s)	Yield % (number of abnormalities/ group of fetuses)
AMC 1,2,3	Karyotyping	0 (0/26)
AMC 1,2,3	Rapid aneuploidy testing	0 (0/46)
AMC 1,2,3	Single gene testing	11.5 (3/26)
AMC 1,2,3	Chromosomal microarray	2.5 (1/40)
AMC 1,2,3	Panel testing	13 (3/23)
AMC 1,2,3	WES	41.7 (10/24)
AMC 1 or 2	All tests	30.8 (4/13)
AMC 3	All tests	27.4 (14/51)

## Discussion

Targeted anomaly scans and motor evaluations in fetuses with multiple contractures, identified during the routine mid-trimester fetal ultrasound examinations, raised the prenatal suspicion of the AMC phenotype in 81 fetuses at our tertiary care center between 2007 and 2021. All parents opted for genetic evaluation before or after birth. Prenatal genetic testing was performed in the majority and combined pre- and postnatal testing in almost a half. A genetic underlying cause was identified in one-quarter of the cases during this 15-year period, increasing to half of the cases during the last 5-year period (2017–2021) due to the introduction of new genetic tests in diagnostics. Among the tests applied, NGS based tests, i.e., (WES based) panel testing and WES had the highest yield (13% and 41.7%).

### Prevalence and phenotypic distribution

The finding of this study concerning the prenatal prevalence of 1.7 per 10,000 is in line with the previously reported live birth prevalence for the different AMC phenotypes. Reported prevalences are 1:10,000 for AMC Group 1 (including Amyoplasia), 1:3000 for AMC Group 2 (with Distal Arthrogryposis), and 1:6985-25,250 for AMC Group 3 (with FADS) [9, 26, 27].

In contrast to the prenatal period, the distinction between the phenotypes of AMC Groups 1, 2, and 3 was enabled postnatally by additional examinations, e.g., neurological examination. This resulted in a postnatal confirmation of AMC Group 1 in 46% (6/13). The low percentage of cases with Amyoplasia in Group 1 of this study (9%, 6/64) differs from prior reported postnatal AMC population, where Amyoplasia is the most frequent type of AMC (25-30% of all individuals with AMC) [1]. A possible explanation is that Amyoplasia (e.g., atrophy) is not easily recognizable in the period of the routine mid-trimester fetal ultrasound examination. Probably, it is easier in the third trimester to recognize this atrophy. All cases with Amyoplasia in our cohort were diagnosed postnatally. Despite improvements in prenatal ultrasound techniques and the expertise of sonographers, it is still challenging to distinguish Amyoplasia and the other types of AMC.

It is emphasized that the fetal phenotypic description was not only supported by anatomical evaluation but also by evaluation of the movements [13, 25, 28]. In general, descriptive motor assessment is mostly applied for routine mid-trimester fetal ultrasound examinations [29]. Despite the term FADS, absent movements are not frequently observed in fetuses with the phenotype AMC Group 3. The “A” in FADS stands for akinesia, which implies the absence of movements. However, based on two studies on consecutive decades of assessing fetal movements in FADS, we have observed that most fetuses with FADS do, in fact, move. In half of the cases, the quantity and differentiation of fetal movements are still within the normal range, while the qualitative performance is abnormal in all [13, 25, 28]. In this study, akinesia was observed in 13 cases, of which once by sMA and twelve times descriptively. The awareness has to remain that in the case of late-onset AMC, the motility does not have to be reduced (yet), even with the effort of serial ultrasound examinations to detect worsening over time.

### Genetic findings

Perinatal genetic testing occurred in all cases of this cohort. However, globally, prenatal genetic testing is not always feasible due to socioeconomic circumstances. The knowledge of the underlying cause is of importance for counseling on the optimal care perinatally. Moreover, parents should be given the opportunity to consider the possibility of termination of pregnancy within the international varying abortion laws, even in case of congenital anomalies.

The overall genetic diagnostic yield in this population increased over the study period from 14% to 50%, largely due to the introduction of NGS-based tests. This yield aligns with other studies in cohorts with AMC demonstrating a genetic diagnostic yield of 35–73% with WES, which is significantly higher than the 5% yield reported for chromosomal microarray [30–40]. This highlights how genetic testing has evolved over the 15-year period with improvements in available genetic tests, and it will continue further when more knowledge is obtained and novel tests are introduced. A new development in the diagnostic pathway is the use of Whole Genome Sequencing (WGS) to improve the diagnostic yield [23]. WGS offers the advantage of detecting a broader range of genetic variations, including copy number variations, structural variants, and non-coding variants, increasing the likelihood of a definitive diagnosis. Thereby, WGS can be used as a single test replacing most of the currently performed tests with a faster turnaround time.

The genetic diagnostic yield in fetuses with the prenatal phenotypes AMC 1 + 2 was 30.8% (4/13). Postnatally, all 4 fetuses were classified as AMC Group 2. Genetic testing in this group revealed one maternally derived chromosomal abnormality, a microduplication 6q24.3q25.2. The mother and maternal grandfather (who also carried the duplication) were affected with mild distal arthrogryposis and had normal cognitive development. The remaining cases had (likely) pathogenic variants in the *ECEL1*, *TGFBR1*, or *PIEZO2* gene. Additionally, there were 2 variants of unknown significance (VUS) in AMC Group 1 + 2, see Supplementary Table 1B. As expected, in the limited number of 6 fetuses with Amyoplasia, no genetic anomaly was identified. Until now, no siblings or descendants have been reported with recurrent Amyoplasia [41]. However, in research settings, there are still ongoing efforts to identify genetic causes for these anomalies [1, 41]. A vascular origin during the embryonic period or epigenetic causes have been suggested [1, 41].

In the AMC Group 3, we found in 27.4% (14/51) of the cases a causal (likely) pathogenic variant. These were in the *ALG12*, *CHRNG*, *DNM2*, *KLHL40*, 2x *MUSK*, *NEB*, *PEX6*, 2x *PIEZO2*, *RYR1*, *SCN4A*, *SLC26A2*, and *TPM2* genes. The majority of these genes are involved in skeletal muscle function (*DNM2*, *KLHL40*, *NEB*, *RYR1*, *SCN4A*, and *TPM2*) in 42.9% (6/14) or the neuromuscular junction function (*CHRNG* and *MUSK*) in 21.4% (3/14) of the cases, which is in line with previous observations [35]. Additionally, there were 5 VUS’s identified in AMC Group 3 cases, see Supplementary Table 1B. Comparable to Group 1 + 2, attention has to be paid to whether these VUS’s could be associated with AMC Group 3 in the future. In one case was no underlying genetic anomaly identified, but the autosomal recessive Neu Laxova Syndrome was clinically suspected.

Among these genes, only three genes, *ALG12, KLHL40*, and *TGFBR1*, have not been listed in Kiefer and Hall’s manuscript, which provides an overview of genes associated with the phenotype AMC in literature until 2019 [2]. *ALG12* has been associated with a congenital disorder of glycosylation, type Ig (OMIM 607143), a phenotype that includes generalized hypotonia, dysmorphic features, and progressive microcephaly, but also musculoskeletal malformations such as overlapping fingers and pes equinovarus have been described [42, 43]. Mutations in *KLHL40* are a frequent cause of severe autosomal-recessive nemaline myopathy with multiple contractures [44]. *TGFBR1* has been reviewed by Baldo et al. in 2022 [45]. They confirmed the association with the AMC phenotype by describing 14 neonatal cases with Loeys-Dietz Syndrome, a phenotype with variable expression of hypotonia and contractures. Furthermore, three *PIEZO2* variants (two with dominant and one with recessive inheritance) were found in this cohort, once (de novo) prenatally suspected as AMC Group 2 and twice (one de novo, one recessive) as AMC Group 3. The diverse phenotypic expression underscores the importance of phenotypic evaluation over time as a goal to evaluate worsening. Autosomal dominant, loss-of-function variants in *PIEZO2* have been associated with the phenotypic overlapping features of Gordon syndrome (DA typ3), Distal Arthrogryposis type 5, and Marden Walker syndrome [46]. The recessive *PIEZO2* variants are associated with loss of function, causing Distal Arthrogryposis with impaired proprioception and touch (DAIPT) [47].

### Strengths and limitations

The strength of this study is the systematic approach to evaluating phenotypic and genotypic characteristics. Tailored prenatal parental counseling by an obstetrician and clinical geneticists was performed and extended through a pediatric physiatrist and orthopedic surgeon in the case of AMC Group 1 + 2 and a pediatric neurologist and neonatologist in the case of AMC Group 3. Our overview of the genetic diagnostic yield over the 15-year period clearly shows the limitations of the genetic tests that were available during the earlier period of the study. On the other hand, the high percentage of DNA storage will facilitate future re-assessment of individual parental requests, potentially not only by WES but also by WGS.

### Recommendations for clinical practice

We highly recommend other centers develop a care pathway in case of prenatal suspected contractures, tailored to the center’s possibilities. A clear step-wise approach supports the multidisciplinary team in planning examinations and counseling [25]. Furthermore, a care pathway stimulates the multidisciplinary awareness to work according to the Human Phenotype Ontology strategy, striving for a precise age-related description of the phenotype and its relation with known pathogenic abnormalities [48–51]. It is also advised to counsel parents about the benefits of WES-based tests, if this test is available. The current study did not reveal any abnormal test results with karyotyping (0/26) or rapid aneuploidy testing (0/46), while chromosomal microarray had only a genetic diagnostic yield of 2.5% (1/40). On the other hand, the chance of identifying a pathogenic chromosomal cause is higher when AMC is associated with multiple structural anomalies [49]. Since nowadays, many DNA diagnostic laboratories offer WES-based CNV testing most of these variants will be identified without the need of additional chromosomal microarray testing. Specific molecular tests in AMC caused by congenital myotonic dystrophies (DMPK) and spinal muscular atrophies (SMA) can be applied [50]. Our population with a modest population size showed that 11.5% (3/26) of the genetic causes were found by single gene testing. In line with our findings, Laquerriere et al. have demonstrated the additional value of WES over panel-based testing in a large cohort of 315 AMC families [35]. This study revealed a genetic diagnosis in 68 of 210 (32%) families. Of the 142 cases without a diagnosis after panel testing, an additional WES was performed in 111 families. In 24 of the 111 (21.6%) families a causal variant was identified by WES. This can be attributed to a wider clinical spectrum of the phenotype for these genes, as well as the identification of novel genes.

## Conclusion

In conclusion, the importance of parental counseling on the possible genetic causes of AMC was highlighted in this 15-year cohort of fetuses suspected of having AMC. Advances in genetic testing techniques during the study period resulted in an increase of the genetic diagnostic yield into half of the cases between 2017 and 2021 due to the introduction of NGS-based tests such as WES. Serial ultrasound examinations are essential to optimize the prenatal detection of AMC due to its variable cause, onset, and expression before birth.

## Supplementary information

Supplementary table 1A and 1B

Supplementary file 2 with latest FADS panel

## Data Availability

All data are presented in Table 1 and Supplementary Table 1. Datasets generated during and/or analyzed during the current study are available from the corresponding author on reasonable request.

## Code availability

All data are presented in Table 1 and Supplementary Table 1. Datasets generated during and/or analyzed during the current study are available from the corresponding author on reasonable request.
