# Supplementary material for: Perinatal genetic diagnostic yield in a population of fetuses with the phenotype arthrogryposis multiplex congenita: a cohort study 2007–2021
Source: Eur J Hum Genet. 2025 Apr 7;34(2):216–26. doi: 10.1038/s41431-025-01848-3 (PMC12859011; doi:10.1038/s41431-025-01848-3)
Supplement: Supplementary file 1 — Supplementary table 1A and 1B [file 41431_2025_1848_MOESM1_ESM.docx]

**Supplementary Table 1A** Prenatal and postnatal phenotype of arthrogryposis multiplex congenita (AMC) classified according to Hall et al., 2014 together with genetic tests and without a pathogenic genetic diagnosis in consecutive cases at Amsterdam UMC during the period 2007-2021. In case of a variance of uncertain significance (VUS), it is presented in Supplementary Table 1B.
* for cases that were seen at location VUmc
** for cases that were included in Tjon et al., 2019^13^

| CASE | PRENATAL | | POSTNATAL | | | Genetic tests  A karyotyping  B rapid aneuploidy  C single gene testing  D chromosomal microarray  E1 panel  E2 WES  Bold for test detecting the mutation | | Pre/postnatal testing?  Pr=prenatal  Po=  postnatal  Pr/Po testing both pre- and postnatal | Pathogenic genetic diagnosis?  If yes: which genetic anomaly  VUS= variant of unknown significance |
| --- | --- | --- | --- | --- | --- | --- | --- | --- | --- |
|  | **Pregnancies with same phenotype** | **Sonographic phenotype**  **Contractures Arms and Legs=CAL**  **Systematic motor assessment= sMA**  **- If yes: differentiation( D),**  **Quality (QL), quantity (QN): normal (nl)/ abnormal (abn).**  **- If no: descriptive**  **AMC Group adapted to Hall et al., 2014:**  **- Group 1+ 2**  **- Group 3** | **Outcome**  **A= alive at birth**  **IUFD= intrauterine fetal death**  **TOP= termination of pregnancy**  **N= neonatal death**  **Gestational age at birth in weeks** | **Phenotype**  **Idem= similar to prenatal phenotype**  **Plus= additional anomalies**  **AMC group according to Hall et al., 2017^4^: 1/ 2/ 3** |  | |  | |  |
| 2007-1 | 1 | CAL: fingers, arms, leg, clubfeet  sMA no, abnormal movements  Other:  abnormal profile, cleft palate,  brain: corpus callosum agenesis, ventriculomegaly,  IUGR and polyhydramnios  AMC 3 | N, 38+5 | CAL: idem plus fracture femur  Other:  Atrophy of upper limbs  Brain idem: corpus callosum agenesis (MRI), plus polymicrogyria  AMC 3 | A | | Pr | | No |
| 2007-2* | 1 | CAL: fingers, hands, elbows, knees, clubfeet  sMA yes 1x, D nl, QL abn, Qn nl  AMC 3 | TOP, 23+5 | CAL idem  Other: dysmorphic features, micrognathia  AMC 3 | A, C | | Pr | | No |
| 2007-3 | 1 | CAL: wrists, clubfeet  sMA: no, absent movements, polyhydramnios  Other: Hydrops fetalis  AMC 3 | TOP, 22+4 | CAL idem,  Other: lung hypoplasia  AMC 3 | A, B | | Pr | | No |
| 2008-2 | 1 | CAL: fixed wrists, clubfeet  sMA: no. Decreased movements  Other: polyhydramnios  AMC 3 | A, 39+6 | CAL idem  Other: retrognathia  AMC 2, distal arthrogryposis | A, C, D, E1 | | Pr/Po | | No, VUS in *MYH8* |
| 2008-4* | 1 | CAL: clubfeet, knees  sMA yes 2x, D nl, QL abn and worsening, QN nl  Other: atrophy legs  AMC 3 | TOP, 23+2 | CAL idem  Other: atrophy legs and retrognathia,  AMC 3 | A, C | | Pr | | No |
| 2009-1 | 1 | CAL: clenched fists, clubfeet  sMA: no. Descriptive decreased movements  AMC 2 | TOP, 22+1 | CAL plus extended knees  AMC 2, distal arthrogryposis | A, B | | Pr | | No |
| 2009-2* | 1 | CAL: wrists, elbows, shoulders, clubfeet, knees, hips  sMA: yes 1x, D, QL, QN abn  Other: micrognathia  AMC 3 | TOP, 22+3 | CAL idem  AMC 3 | A, C | | Pr | | No |
| 2009-3*, ** | 6 | Flexed spine  sMA: yes, D, QL and QN abn and worsening over time  Other: placental thickening and multiple cysts. See case report in Tjon et al., 2021^47^  AMC 3 | IUFD 6x at 21+0, 21+0, 22+2, 24+1, 21+6 and 20+5 | Flexed spine idem, plus contractures from shoulders to ankles  Others: facial flattening  profile, mild retrognathia  Massive Perivillous Fibrin Deposition  AMC 3 | A, C, E2 | | Pr/Po | | No, VUS in *VARS1* and *ABCF1* |
| 2010-2* | 1 | CAL: fingers, clubfeet  sMA: yes, 2x D, QL and QN nl  Other: polyhydramnios  AMC2/ 3 | A, 33+3 | CAL idem  Plus submucous, cleft palate  Nemaline myopathy, genetically not confirmed  AMC 2/3 | A, B | | Pr | | No |
| 2010-3* | 1 | CAL: wrists, clubfeet  sMA: no, decreased movements  Other: Hydrops, choroid plexus cysts  AMC 3 | TOP, 21+2 | CAL idem  Other: hydrops  AMC 3 | A, B, C, D | | Pr | | No |
| 2010-4* | 1 | CAL: wrists, clubfoot  sMA: no, abnormal movements  Other: hydrops  AMC 3 | TOP, 18+2 | CAL plus hips and knees  Other: dysmorphic features, micro/retrognathia  Spina bifida occulta  AMC 3 | A, C | | Pr | | No |
| 2010-5* | 1 | CAL: elbows extended, wrists flexed,  sMA : yes 1x. D abn no participation elbow, wrist, fingers QL abn, idem QN nl.  AMC 1+ 2 | TOP, 23+3 | CAL idem,  other: retrognathia, possibly webbing armpits  AMC 1, Amyoplasia | A, C, D | | Pr | | No |
| 2010-6 | 1 | CAL: knees, hips, wrists  sMA: no. Descriptive: no movements in knees and hips  Other: retrognathia, polyhydramnios  AMC 3 | A, 37+4 | CAL plus shoulders, elbows, wrists  AMC 2, Distal Arthrogryposis | D, E1 | | Po | | No. VUS in *MYH8* |
| 2011-1* | 1 | CAL: wrists, clubfeet  sMA: D nl, QL abn and worsening over time, QN nl    AMC 3 | TOP, 17+0 | CAL plus elbow and knees,  Other: atrophy extremities, retro/micrognathia  AMC 3 | A, C | | Pr | | No |
| 2011-2 | 1 | CAL: elbows, wrists, left hip luxated, leg fixed and extended, right knee flexed  sMA: no. Absent fetal movements  Brain: ventriculomegaly  AMC 3 | TOP, 23+2 | CAL plus fingers and clubfeet. Plus:  retro/micrognathia  Brain: focal disturbances of cortical layers  AMC 3 | A, B, C, D | | Pr/Po | | No |
| 2011-3* | 1 | CAL: elbows, wrists, knees  sMA: yes 2x. D nl, QL abn and worsening over time, QN Mouth open during whole examination.  AMC 3 | TOP, 22+2 | CAL idem  Other: Webbing knee and atrophy, retrognathia, polydactyly foot  AMC 3 | A, B, C | | Pr | | No |
| 2011-4 | 1 | CAL: hands, wrists, elbows, clubfeet  sMA no, slow movements  Other: echogenic bowels, dolichocephaly  AMC 3 | TOP, 23+5 | CAL idem  AMC 2/3 | A, B, C | | Pr | | No |
| 2012-1* | 1 | CAL: wrists and clubfeet  Other: atrophy arms  sMA: yes 2x D abn, no arm movements, QL nl except for restricted participation arms, QN nl,  AMC 1+ 2 | A, 39+3 | CAL plus elbows and shoulders  AMC 1, Amyoplasia | D | | Pr | | No |
| 2012-2* | 1 | CAL clubfeet, oligodactyly both hands,  sMA no, absent fetal movements  Other: hydrops, scoliosis  AMC 3 | TOP, 20+1 | CAL plus syndactyly (no oligodactyly)  Other: webbing, cleft palate Plus: arachnodactyly, micro/retrognathia, and hypoplastic lungs  AMC 3 | A, C, D | | Pr | | No |
| 2012-4 | 1 | CAL elbows, wrists, knees, clubfeet.  sMA no, abnormal movements  Other: echogenic bowels, micrognathia, enlarged nuchal fold  Brain: microcephaly, abnormal profile, corpus callosum agenesis, hypoplastic cerebellum  AMC 3 | N, 40+0. Child died after a hour | CAL plus hips  Other: Exophthalmia, short limbs, cleft palate  AMC 3/ Neu Laxova | A, B, D | | Pr/Po | | No,  Autosomal recessive Neu Laxova based on phenotype |
| 2012-6 | 2 | CAL arms, overlapping fingers,  sMA no, absent movements,  Other: hydrops, polyhydramnios  AMC 3 | N, 39+5  IUFD, 37+5  First child died due to respiratory problems caused by hypotonia | CAL idem  Other: cleft palate in first child  AMC 3 | A, B, C, E1, E2 | | Pr/Po | | No |
| 2012-7* | 1 | CAL: wrists, clubfoot  sMA: yes 2x, stable, D nl, QL abn both arms. QN nl  Other: short femur  AMC 3 | TOP, 23+2 | CAL plus elbows, wrists, knee and hips, clubfeet  Other: micro/retrognathia, hypertelorism, median cleft palate, horseshoe kidney  AMC 2/ 3 | B, D, E1, E2 | | Pr | | No |
| 2012-8* | 1 | CAL: wrists, clubfeet  sMA yes 1x, D nl, QL abn, QN nl  AMC 3 | TOP, 20+0 | CAL plus elbows, shoulders, wrists  Other: webbing, mild facial dysmorphias  AMC 3 | B, C, D, E1 | | Pr | | No |
| 2012-9*, ** | 1 | CAL: wrists, clubfeet  sMA yes 2x, D abn , QL abn, abrupt and fluent movements, QN nl  Other: enlarged nuchal translucency, short femur  Brain: small cerebellum, periventricular cysts, gyration reduces  AMC 3 | N, 39+6  Died after 3 hours due to respiratory dysfunction | CAL plus shoulders and finger  Other: abnormal profile with retrognathia  Brain: microcephaly with polymicrogyria, initiated by early germinal matrix bleeding with periventricular cysts  AMC 3 | A, B, C, D, | | Pr | | No |
| 2013-1 | 1 | CAL: elbows, knees, clubfeet  sMA: no, abn movements  Other: abnormal profile  AMC 3 | TOP, 22+3 | CAL idem  Other: micrognathia  AMC 3 | A, B, C, D | | Pr/Po | | No |
| 2013-2* | 1 | Clubfeet  sMA: yes 1x, D nl, QL abn, QN nl  AMC 3 | TOP, 23+6 | CAL plus  elbows, hips and knee  AMC 3 | B, D, E1 | | Po | | No |
| 2013-3* | 1 | CAL: elbows, wrists  sMA yes 2x, D and QL abn, QN nl  AMC 3 | TOP , 15+5 | CAL plus shoulders and fingers, hypotrophy muscles arms and shoulders, micrognathia  AMC 3 | B, C, D, E1, E2 | | Pr/Po | | No |
| 2014-1 | 1 | CAL: wrists, fingers  sMA: no. Movements present in trunk, not in elbows, knees, wrists and fingers  Other: micrognathia, intrauterine growth restriction p2  AMC 1+ 2 | A, 39+1 | CAL idem  Other: mild retrognathia  AMC 2, distal arthrogryposis | D, E1 | | Po | | No |
| 2015-1 | 1 | CAL: wrists, clubfeet  sMA: no, abnormal movements  Other: oligohydramnios  AMC 3 | TOP, 13+5 | CAL idem  Other: Micrognathia, webbing  AMC 3 | B, D, E1, E2 | | Pr/Po | | No |
| 2015-3* | 1 | CAL: wrists, elbows, clubfoot  sMA: yes. 2x D, QL and QN nl. Decreased participation   AMC 1+ 2 | TOP, 16+5 | CAL idem  AMC 1, Amyoplasia | B, D, E1, E2 | | Pr | | No  VUS in *NEB* |
| 2015-4* | 1 | CAL: shoulders, elbows, wrists, hips, knees, ankles  sMA yes, absent fetal movements  Other: polyhydramnios  AMC 3 | TOP, 35+3 | CAL plus fingers  Other: Micro/retrognathia, and Tent-shaped mouth  AMC 3 | D, E2 | | Pr/Po | | No |
| 2016-1* | 1 | CAL: overlapping fingers, clubfeet  sMA: yes 1x, D nl, QL abn due to decreased variability in isolated arm-, leg movement and general movements. QN nl  Other: oligohydramnios  AMC 1+ 2 | A,38+5 | CAL idem  AMC 2, Distal Arthrogryposis | E2 | | Po | | No, VUS in *MYH3* |
| 2016-2 | 1 | CAL: clinodactyly hands bilateral, clubfoot left, fixed wrists bilateral  Intrauterine growth restriction p1.  sMA: no. Descriptive normal  AMC 1+ 2 | A, 41+5 | CAL idem  AMC 1, Amyoplasia | B, D, E1 | | Pr/Po | | No |
| 2016-4*, ** | 1 | CAL: fingers, clubfeet, knees  sMA yes, D and QL abn, QN nl  Other: polyhydramnios, small thorax  AMC 3 | TOP, 21+3 | CAL idem  Other: abnormal profile, retro/ micrognathia, cleft palate  AMC 3 | B, C, E2 | | Pr/Po | | No |
| 2017-3*, ** | 1 | CAL fingers, knees, clubfeet  sMA yes 1x, D and QN nl, abn QL.  Other: retrognathia,  Brain: hypoplastic cerebellum, corpus callosum agenesis,  Heart: tetralogy of Fallot  IUGR  AMC 3 | TOP, 23+3 | CAL idem  Other: hypertelorism, cleft palate, retrognathia, hypospadias, webbing  AMC 3 | B,D,E2 | | Pr | | No |
| 2017-5 | 1 | CAL: elbows, wrists, knees, clubfeet  SMA: no, abnormal movements  AMC 3 | TOP, 22+5 | CAL plus fingers  AMC 2/3 | B, D | | Pr | | No |
| 2017-7*, ** | 3 | CAL: fingers, wrists, clubfeet  sMA: no. Minimal/no fetal movements  Other: hydrops  Heart: truncus arteriosus  AMC 3 | 2x IUFD, 27+6 and 24+2  1x TOP, 14+5 | CAL idem/ plus.  Hydrops idem  other: webbing, dysmorphic features (hypertelorism), cleft palate  AMC 3 | B, D, E2 | | Pr | | No, VUS in *RYR1* |
| 2018-1* | 1 | CAL: overlapping fingers, clubfeet  sMA: no. Descriptive normal movements  Other:  micrognathia, polydactyly, echogenic kidney, cleft palate  Heart: DORV and VSD,  Single umbilical artery  AMC 3 | TOP, 16+6 | CAL idem*,*  Other: dysmorphic features (large forehead, small mouth, low ears, retrognathia)  AMC 2/3 | B, C, D, E2 | | Pr/Po | | No |
| 2018-3 | 1 | Fixed elbows and wrists  sMA: no, descriptive normal movements  AMC 1+ 2 | A, 40+3 | Idem  AMC 1, Amyoplasia | B, D, E2 | | Pr | | No |
| 2018-5*, ** | 1 | CAL: fingers, wrists, elbows and clubfeet  sMA yes, D nl, QL abn nl, QN nl  Other: liver cysts  AMC 3 | TOP, 16+6 | CAL plus hips  Other: dysmorphic features  AMC 3 | B, D | | Pr | | No |
| 2019-6 | 1 | CAL: hands, fixed elbows and wrists, clubfeet  sMA: no. No descriptives  AMC 1+ 2 | TOP,21+3 | CAL plus knees  Other: webbing right armpit, atrophy arms  AMC 1, Amyoplasia | B, D, E1 | | Po | | No |
| 2020-2 | 1 | CAL: wrists, hips, clubfeet  sMA no, decreased movements  Other:  Small thorax, sacral agenesis  AMC 3 | TOP, 17+6 | CAL plus fingers, wrists  Other: hypertelorism, retrognathia scoliosis, webbing elbow and agenesis extended to thoracal spine. Mother no known diabetes Mellitus  AMC 3 | B, D | | Pr | | No |
| 2020-4* | 1 | CAL: fingers, clubfeet  sMA yes 2x, D and QL abn, QN nl  Other: micrognathia,  empty stomach, polyhydramnios  AMC 3 | N, 36+6  Died 10 days after birth due to respiratory insufficiency | CAL idem  Other: dysmorphic features, cleft palate,  Brain: pons, cerebellum, mesencephalon, hippocampi abnormal development, hypertelorism  AMC 3 | B, D, E1 | | Po | | No |
| 2020-6* | 1 | CAL: clubfoot right, bilateral extended knees  sMA yes 2x, D nl, QL abn and worsening over time, QN nl  AMC 3 | IUFD, 24+4 | CAL: idem  Other: dysmorphic features, short limbs  AMC 3 | B, E1 | | Pr/Po | | No |
| 2020-7 | 1 | CAL: elbows and knees  sMA no, absent fetal movements  Other: hydrops  AMC 3 | TOP, 12+5 | D&C interfered post-mortem examination, inclusion based on severity of prenatal findings  AMC 3 | B, C, E1, E2 | | Pr | | No |
| 2021-1 | 1 | CAL: fingers, wrist, knees, clubfeet  sMA: no, minimal movements  AMC 3 | TOP, 23+5 | CAL plus elbows  Other: Micrognathia, hypertelorism  AMC 3 | B, E1 | | Pr/Po | | No |

**Supplementary Table 1B** description of genotype based on perinatal genetic tests of 6 fetuses with a variance of uncertain significance (VUS) and AMC identified in the prenatal period at Amsterdam UMC during the period 2007-2021. Classification following ACMG Criteria^52^.

| CASE | Inheritance  DN = de novo  AR = autosomal recessive | Nucleotide alteration, deduced protein change | ACMG criteria  P=pathogenic  LP=likely pathogenic | Aggregated Pathogenicity Prediction (Franklin) | MAF (according to gnomAD v4.1.0) |
| --- | --- | --- | --- | --- | --- |
| 2008-2 | Heterozygous (maternal) | NM_002472.3(MYH8):c.4738G>A p.(Ala1580Thr) Chr17(GRCh37):g.10298674C>T | VUS: PM2 | Uncertain (0.35) | 0.00008178 |
| 2009-3* | Homozygous, both parents heterozygous | NM_006295.3(VARS1):c.518G>T p.(Arg173Leu) Chr6(GRCh37):g.31760767C>A  NM_001025091.1(ABCF1):c.1510G>A p.(Val504Ile) Chr6(GRCh37):g.30553369G>A | VUS: PM2  VUS: PM2 | Uncertain (0.31)  Uncertain (0.68) | 0.000009915 (no homozygous alleles)  0.00002356 (no homozygous alleles) |
| 2010-6 | Heterozygous (maternal) | NM_002472.3(MYH8):c.3025A>G p.(Thr1009Ala) Chr17(GRCh37):g.10304675T>C | VUS: PM2 | Uncertain (0.54) | - |
| 2015-3* | Heterozygous (paternal) | NM_001271208.2(NEB):c.22475G>C p.(Ser7492Thr) Chr2(GRCh37):g.152381033C>G | VUS: PM2 | Uncertain (0.59) | 0.0005756 (1x homozygous) |
| 2016-1* | Heterozygous (paternal) | NM_002470.4(MYH3):c.1495C>G p.(Gln499Glu) Chr17(GRCh37):g.10546229G>C | VUS: PM2, PP3 | Deleterious (0.73) | - |
| 2017-7*,** | Heterozygous (paternal) | NM_000540.3(RYR1):c.12945_12947del p.(Arg4316del) Chr19(GRCh37):g.39055919_39055921del | VUS: PM2, BP3 | Benign (0.01) | 0.000002009 (no homozygous alleles) |
