# Supplementary material for: Perinatal genetic diagnostic yield in a population of fetuses with the phenotype arthrogryposis multiplex congenita: a cohort study 2007–2021
Source: Eur J Hum Genet. 2025 Apr 7;34(2):216–26. doi: 10.1038/s41431-025-01848-3 (PMC12859011; doi:10.1038/s41431-025-01848-3)
Supplement: Supplementary file 2 — with latest FADS panel [file 41431_2025_1848_MOESM2_ESM.docx]

**Supplementary File 2.** Overview of genes that are included in the FADS panel of Amsterdam UMC in 2025

- ACTA1
- ACVR1
- ADCY6
- ADGRG6
- AGL
- AGRN
- ALG3
- AMER1
- ANO5
- ASCC1
- ATP1A3
- ATP2A1
- ATP7A
- B3GALNT2
- B4GAT1
- BAG3
- BICD2
- BIN1
- CACNA1S
- CACNB2
- CACNB2
- CAPN3
- CASQ1
- CAV3
- CAVIN1
- CCDC78
- CFL2
- CHAT
- CHCHD10
- CHKB
- CHRNA1
- CHRNB1
- CHRND
- CHRNE
- CHRNG
- CHST14
- CLCN1
- CNTN1
- CNTNAP1
- COL12A1
- COL13A1
- COL6A1
- COL6A2
- COL6A3
- COLQ
- CPT2
- CRYAB
- DAG1
- DENND5A
- DES
- DHCR24
- DMD
- DMPK
- DNA2
- DNAJB6
- DNM2
- DNM3
- DOK7
- DPAGT1
- DPM1
- DPM2
- DPM3
- DYNC1H1
- DYSF
- ECEL1
- EGR2
- EMD
- ENO3
- ERBB3
- ERCC5
- ERCC6
- EXOSC8
- FAM111B
- FAM20C
- FBN2
- FGFR2
- FHL1
- FKBP14
- FKRP
- FKTN
- FLNC
- FOXP3
- GAA
- GBA
- GBE1
- GFPT1
- GLDN
- GLE1
- GNE
- GYS1
- HNRNPH1
- HSPG2
- IGHMBP2
- IRF6
- ISCU
- ISPD
- ITGA7
- KBTBD13
- KCNJ2
- KLHL40
- KLHL41
- KLHL9
- LAMA2
- LAMB2
- LAMP2
- LARGE1
- LDB3
- LDHA
- LMNA
- LMOD3
- LPIN1
- MAGEL2
- MATR3
- MEGF10
- MICU1
- MPZ
- MSTN
- MTM1
- MTMR14
- MUSK
- MYBPC1
- MYBPC1
- MYBPC2
- MYF6
- MYH2
- MYH3
- MYH7
- MYH8
- MYOD1
- MYOT
- NEB
- NEK9
- ORAI1
- PABPN1
- PDHA1
- PFKM
- PGAM2
- PGK1
- PGM1
- PHKA1
- PIGH
- PIGH
- PIP5K1C
- PLEC
- PNPLA2
- POMGNT1
- POMGNT2
- POMK
- POMT1
- POMT2
- PRPS1
- PYGM
- RAC1
- RAPSN
- RBCK1
- RHEB
- RIPK4
- RRM2B
- RXYLT1
- RYR1
- SCN4A
- SELENON
- SGCA
- SGCB
- SGCD
- SGCG
- SLC52A2
- SLC52A3
- SMCHD1
- SMN1
- SPEG
- STIM1
- STX1B
- SYNE1
- TANGO2
- TCAP
- TNNI2
- TNNT1
- TNNT3
- TNNT3
- TNPO3
- TPM2
- TPM3
- TRAPPC11
- TRIM32
- TRIP4
- TRPV4
- TTC19
- TTN
- UBA1
- UTRN
- VAMP2
- VCP
- VIPAS39
- VMA21
- VPS33B
- VRK1
- ZBTB33
